# Experimental Designs for Preclinical Neuroscience Experiments: Part I—Design Basics

**DOI:** 10.1523/ENEURO.0007-26.2026

**Published:** 2026-02-24

**Authors:** P. S. Reynolds

**Affiliations:** University of Florida, Gainesville, Florida 32610

**Keywords:** 3Rs, design of experiments, randomization, replication, reproducibility, variance minimization

## Abstract

Rigorous, statistically grounded experimental design is central to ethical and effective animal research. Foundational principles for statistically based Design of Experiments (DOE) were established over a century ago by Sir Ronald Fisher. They have since been augmented by modern computational tools that now enable researchers to implement designs that maximize scientific information and benefit while minimizing harms. However, many preclinical investigators are unfamiliar with formal DOE methods. Poorly designed experiments followed by inappropriate statistical analyses contribute to poor reproducibility, translational failure, and unnecessary animal use. This first paper in a three-part series introduces neuroscience researchers to the fundamentals of statistically based experimental design as a substitute for traditional two-group comparisons. Key components of a designed experiment are defined, along with the importance of correctly identifying experimental units to avoid pseudo-replication. Fisher's three essential design principles—randomization, replication, and blocking—are presented as nonoptional practices for controlling bias, managing variation, and ensuring valid statistical inferences. Particular emphasis is placed on probability-based random allocation, the use of validated computer-generated randomization plans, and the role of blocking in reducing nuisance variation. By embedding robust design principles early in study planning, researchers can produce reliable, reproducible, and ethically justifiable data. Subsequent papers in the series will expand on methods for controlling unwanted variation through blocking (Part 2) and outline flexible multivariable design strategies (Part 3). Worked examples and R code are included.

## Introduction

In their groundbreaking book *The Principles of Humane Experimental Technique*, William Russell and Rex Burch articulate the pillars of what are now known as the 3Rs of animal research: Replacement, Reduction, Refinement. Central to their arguments is the concept of a well-designed experiment. A “good” experiment meets the 3Rs objectives by providing the “maximum amount of scientific information for minimal harms” (Russell and Burch, 1959). The criteria for constructing an informative experiment are primarily statistical, belonging to a specialized area of applied statistics known as Design of Experiments (DOE). Pioneered in the 1920s by Sir Ronald Fisher, DOE includes some of the most important and influential statistical ideas ever proposed (Yates and Mather, 1963; Rao, 1992). The methods developed by Fisher have been greatly extended since they were first developed over a century ago. Advances in computational speed and memory, software development, and computer graphics capabilities now provide researchers with far more powerful, easily applied, and user-friendly tools than were previously available.

A well-designed experiment depends on statistically based best practice methods for structuring data collection. These methods ensure that the data produced from the experiment are statistically valid and informative for the least and most efficient use of limited resources. This means that results are reliable, valid, reproducible, and above all ethical (Altman, 1980). Regrettably, most preclinical researchers (and even many consulting statisticians) are completely unaware of even the most basic principles of formal experimental design. Poorly designed studies contribute to the well documented failures in reproducibility and translation for much animal-based research resulting in the waste of countless study animals in noninformative and even misleading or harmful experiments (Johnson, 2021; Wold and Tabak, 2021).

This paper is the first of a three-part series providing a basic introduction to statistically based design of experiments for neuroscience researchers. Part 1 outlines the advantages of a well-designed experiment and covers DOE basics and terminology. Part 2 shows how unwanted variation can be minimized or controlled by blocking, a fundamental of DOE. Finally, Part 3 describes five broad design categories that demonstrate different customizable strategies for the efficient and economical evaluation of multiple predictor variables in a single study. Worked examples are provided together with R code for design construction and random allocation.

This series is by no means an exhaustive coverage of the topic of experimental design. The reader is advised to refer to resources and references listed at the end of each article in this series.

## Time to Phase Out the “Two-Group” Study

Two-group experiments are still the norm in neuroscience literature. Studies typically consist of a group of animals assigned to an experimental (or test) condition and compared with those of a second group assigned to the control condition. However, two-group experiments are poorly suited to preclinical research. Numerous redundant control groups waste animals without increasing usable information. Forcing comparisons between only two groups results in the researcher being unable to detect biologically important patterns and interactions that would become apparent when multiple variables are considered simultaneously in a designed study.

## Advantages of the Designed Multivariable Experiment

Compared with the two-group approach, a designed experiment is more flexible, efficient, and ultimately sparing of animals and resources. Properly designed experiments can accommodate the simultaneous investigation of multiple explanatory variables, identify which variables have the greatest effects on the response, and quantify patterns of relationships between variables (synergism, inhibition). Formal statistically structured experimental designs have the statistical property of orthogonality; that is, the effect of each of several factors can be isolated and estimated separately so that the results of the analysis are more easily interpreted. Design methods such as randomization and blocking are fundamental for minimizing bias and controlling unwanted sources of variation. Even if the results cannot deliver a clear-cut decision as to whether or not the experiment “worked,” a well-designed experiment will provide enough actionable information to guide the design of subsequent experiments (Czitrom, 1999).

## Components of a Designed Experiment

The three major components of a designed experiment are *inputs*, *outputs*, and *experimental units*. To ensure the experiment is robust, unbiased, and efficient, Fisher also advocated incorporation of three essential design principles: *randomization*, *replication*, and *blocking*.

### Input variables

The input variables are the independent or predictor variables manipulated by the researcher. In a designed experiment, they are referred to as *factors*. Each factor consists of two or more prespecified values or *factor levels*. The factor levels can be categorical (as in a nominal, binary, or ordinal descriptor) or quantitative (as in a measurement). A *treatment* is one of each combination of factor levels. Treatments are randomly assigned by the researcher to the individual *experimental units* (see below). An experimental *group* consists of all experimental units receiving the same treatment.

*Example*. A researcher wants to study the effects of a certain drug administered either with or without a specific antibiotic on male and female mice. The researcher plans to assess the effects of two doses of the drug (5 mg/ml; 15 mg/ml) against vehicle control and a fixed dose of the antibiotic (5 mg) versus vehicle control.

There are three factors Drug, Sex and Antibiotic. Factor Drug has three factor levels: zero dose (vehicle control), low dose (5 mg/ml), and high dose (15 mg/ml). Factor Sex has two categorical levels, male and female. Factor Antibiotic has two levels which can be considered as either categorical (present, absent) or quantitative (5 mg, 0 mg). There are therefore 3 × 2 × 2 = 12 treatments (Table 1).

The number of factors and levels for each factor determine the number of possible treatment combinations that can be tested and therefore the specific design of the experiment. Choice of factor levels is determined by preliminary data or literature values that indicate a reasonable range to bracket the possible response and a consideration of practical logistic constraints such as cost or resource limitations. For screening experiments, when the objective is to narrow down a large candidate pool of potential factors, two levels are sufficient. For determining an optimum, identifying a target response, or assessing complex nonlinear relationships, designs with relatively few factors and three or more levels per factor are more effective.

**Table 1.** Treatments for a three-factor experiment with factors drug, antibiotic, and sex, with each factor at two levels

Drug	Antibiotic	Sex
High	Present	Male
Low	Present	Male
None	Present	Male
High	Absent	Male
Low	Absent	Male
None	Absent	Male
High	Present	Female
Low	Present	Female
None	Present	Female
High	Absent	Female
Low	Absent	Female
None	Absent	Female

There are 12 possible treatments with all possible combinations of factors.

### Output variables

The outputs of a designed experiment are the *outcome*, *response*, or *dependent* variables. Outcome variables are measured to determine the effects of the experimental manipulations; that is, if the experiment “works.” All outcome variables for a study must be prespecified, clearly defined, and measurable. Outcome metrics that are vaguely defined cannot be measured and therefore cannot be adequately assessed as a test of the research hypothesis.

The “best” outcome variables will be the most informative, that is they will be the most relevant to the research question, large enough to be conveniently measured, consistent, and stable. During study planning, all potential outcome variables should first be prioritized and ranked according to their relevance to the research objectives and their feasibility. The *primary outcome* is the most important as it is the basis for power calculations and interpretation of results. Lower-priority variables provide supporting information but do not determine sample size and cannot provide a definitive test of the research hypothesis. Although researchers usually want to measure as many outcomes as possible, indiscriminate hypothesis testing on every and all outcome variables to find “statistical significance” greatly increases the chances of finding false positive results (Type I error), muddies interpretation, and leads to questionable research practices such as P-hacking and data dredging (Altman and Krzywinski, 2017).

The *type* of outcome variable must also be considered as it determines both sample size and subsequent choice of statistical tests. Binary variables have only two possible outcomes (yes/no; present/absent). Time to event variables measure the amount of time between a defined starting point to a specific, important event (like disease recurrence or death) and are central to survival analysis. Both types require very large sample sizes for adequate power and common statistical analysis methods appropriate for continuous outcomes data such as *t* tests cannot be used (Altman, 1991). In contrast, continuous outcome variables have the greatest statistical power for a given sample size and require a smaller sample size to detect a specific effect size. Therefore, in accordance with the 3Rs, planning priority should be given to continuous over binary or time to event outcome variables because sample sizes required for testing hypotheses are smaller. Choice of outcome variables must also account for humane endpoints. These are study-relevant welfare indicators that determine when the animal will be removed from the study (Reynolds, 2024).

### Experimental units

The *experimental unit* is defined as the smallest independent entity that can be *independently assigned a specific treatment* (Lazic et al., 2018; Huang et al., 2020). The experimental unit should not be confused with the *observational unit* which is the smallest independent entity from which *measurements* are obtained. The number of experimental units is the total sample size.

**Figure 1.**
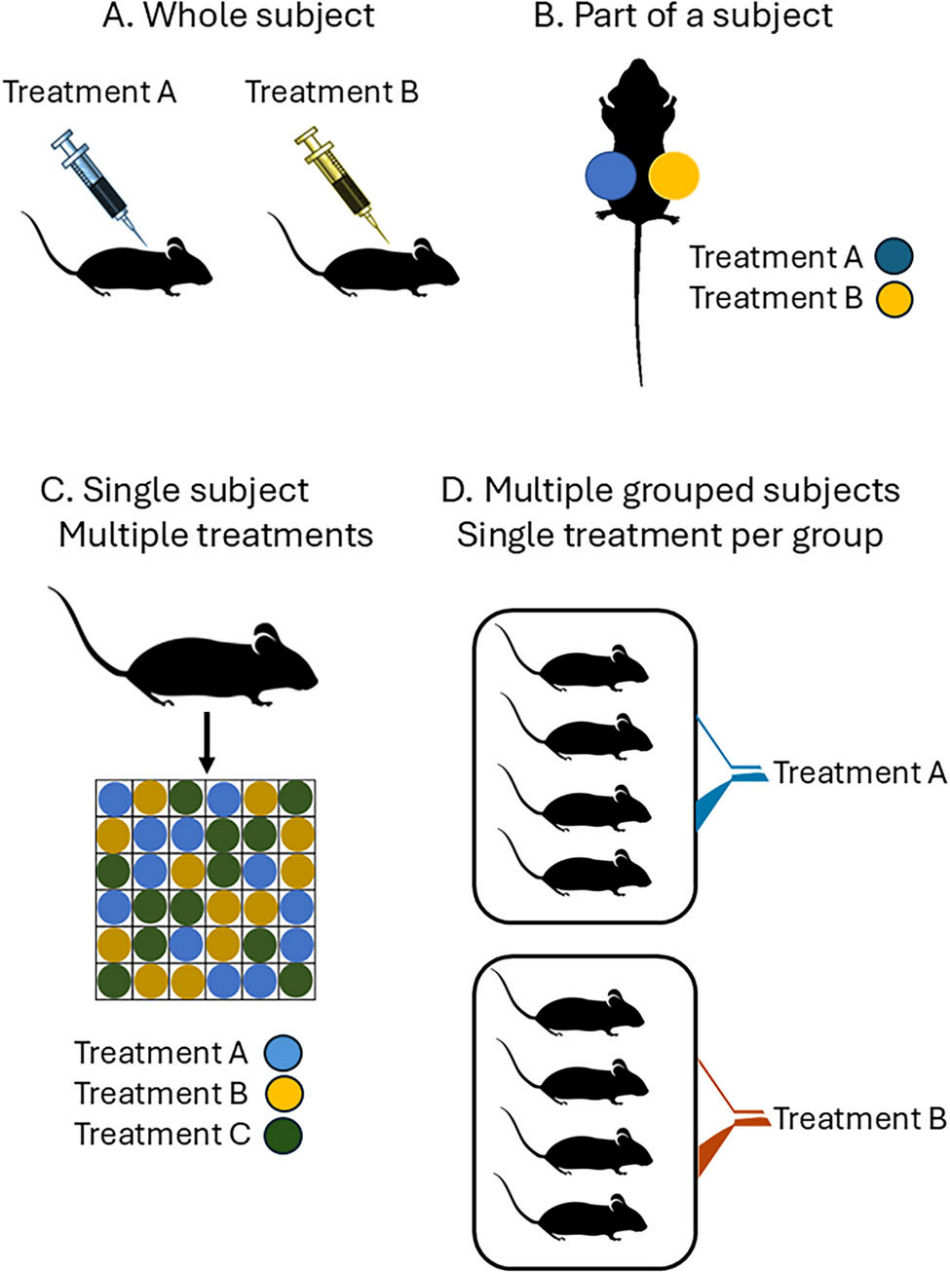
Examples of experimental units. Color codes indicate separate treatments. ***A***, The experimental unit is the whole subject. Here each mouse receives a separate treatment intervention (drug injections), and the individual mouse is the experimental unit. The individual is also the biological unit. ***B***, The experimental unit is each flank of a mouse. Treatment A is randomized to either the right or left flank of each mouse and the second treatment is applied to the opposite flank. ***C***, The experimental unit is each individual well of the plate: three different drugs are applied to each well independently according to a Latin Square design. The individual mouse is the biological unit. Individual mouse identity can be incorporated as a block factor if whole animal effects are not of direct interest, but it is desired to control unwanted between-mouse variation. ***D***, The experimental unit is the cage: in one cage all four mice receive drug A, and four mice in the second cage all receive drug B. Individual mice are biological units. The total sample size is *N* = 2 (not 8), because there is only one experimental unit per treatment group. Analyzing the data as if they represent eight independent experimental unit (*n* = 4/group) is pseudo-replication.

Categories of experimental unit are shown in Figure 1. This shows situations where the experimental unit is not necessarily the individual animal. It is important to correctly identify the experimental unit during the planning stages of the study and before data are collected and analyzed. Analysis of nonindependent observations as if they are independent experimental units is *pseudo-replication*. The consequences of misidentifying the experimental unit include artificial inflation of the sample size, increase in false positives, and serious compromise or invalidation of statistical analyses (Lazic et al., 2018).

### Fisher’s three essentials

Fisher considered randomization, replication and blocking to be requirements, not options, for reliable experimentation. These three methods are fundamental to addressing problems central to all experimentation: bias and confounding due to unknown factors, unwanted variation, and experimental noise. Their inclusion maximizes the accuracy and reliability of results (study *internal validity*) for the least expenditure of resources, time, and animals. Unfortunately, many researchers are either unaware of these methods or regard them as either unnecessary or inconvenient (Hall, 2007).

#### Randomization

*Randomization* is the formal probability-based process of assigning treatments to experimental units. When experimental units are processed sequentially, the order of experimentation should also be randomized (random sequence allocation). The ARRIVE 2.0 guidelines identify randomization as one of the Essential Ten items to be reported in published research involving animals (Percie du Sert et al., 2020).

Properly performed randomization ensures the validity of statistical hypothesis tests. It protects against confounding of true differences between treatments (which we want to detect) with spurious differences resulting from selection bias, systematic bias, and unanticipated trends in the data. It reduces the effects of natural variation that occurs both between and within animals, increasing precision of the treatment difference estimates. Finally, it ensures that results of statistical tests are robust, efficient, and valid so that interpretation will be reliable (Preece, 1990; Bespalov et al., 2019).

There are four steps to performing random allocation:

1. Identify the experimental unit (the unit of randomization).
2. Identify the treatments (factors and the number of levels of each factor).
3. Identify the formal study design (e.g., completely randomized, randomized complete block, factorial, nested, split plot, etc.).
4. Generate a replicable allocation schedule (e.g., in R code).

There are two provisos to correctly performing random allocation. First, *the randomization plan must be tailored to the design*. The simplest method of randomization as understood by many researchers is *simple random allocation* where each experimental unit has equal probability of receiving any study treatment. However, this method does not account for the experiment structure, and if the total sample size is small (which is usually the case for animal studies), it is extremely liable to chance imbalances in sample size between treatment groups. It is therefore not recommended for animal studies. *Restricted randomization* is another simple randomization method that is constrained to generate equal sample sizes for the treatment groups. However, more complex design structures will require more efficient randomization plans to take advantage of the study design features. For example, block designs group similar experimental units into “blocks” based on some common feature (the blocking or nuisance factor). Randomization is performed separately for each block. Blocking not only ensures sample size balance across treatments, it also controls unwanted variation associated with that factor. In contrast, hierarchical designs such as split-plot have levels of one treatment factor nested in the levels of a second factor. Therefore, randomization must be conducted in two stages, first for the allocation of the primary treatments to the main factor experimental units, then for allocating the secondary nested treatments to the subfactor units.

Second, *the randomization plan must be generated with a validated*, *computer-based algorithm*. Seed numbers for the randomization procedure ensure reproducibility and provide an audit trail. Randomization plans for complex experimental designs such as optimal designs and balanced incomplete block designs can only be conveniently constructed by computer algorithms. Obsolete methods based on coin tosses, dice rolls, or random number tables are not recommended. They cannot be reproduced and are easily manipulated by the investigator. In the next two articles, worked examples accompanied by R code will be provided to demonstrate how random allocation plans can be customized for specific designs.

Although the role of randomization in study design has been extensively addressed for decades, regrettably it is not yet standard practice for most animal experiments (Kilkenny et al., 2009; Hirst et al., 2014), and both the concept and purpose are still widely misunderstood. Researchers often confuse “random” with “unplanned” or “haphazard” allocation. Sequential, serpentine, quasi-random, alternating, or other non-random allocation strategies are often described incorrectly as “random.”

#### Replication

*Replication* in the context of experimental design is the number of independent observations required to estimate the within-group or unexplained variation in the ANOVA model. It is used to evaluate statistical significance of the treatment differences. For balanced single-factor completely randomized designs and randomized complete block designs, the number of replicates will be the sample size per treatment group *n* (Doncaster and Davey, 2007). With more complex designs, the effective sample size *n* will depend on how measurements are distributed according to the structure of the design (Blainey et al., 2014). For example, for a balanced factorial design with three independent factors A, B, and C with levels *a*, *b*, and *c*, respectively, the number of replicates for the main effect of factor A will be *bcn*, the number of replicates for the two-way interaction A × B will be *cn*, and for the three-way interaction A × B × C will be *n* (Doncaster and Davey, 2007). This example also demonstrates the greater efficiency of the factorial design compared with the single factor (“one-way ANOVA”) layout; that is, the number of replicates for each factor is greatly increased without increasing the total number of animals.

**Figure 2.**
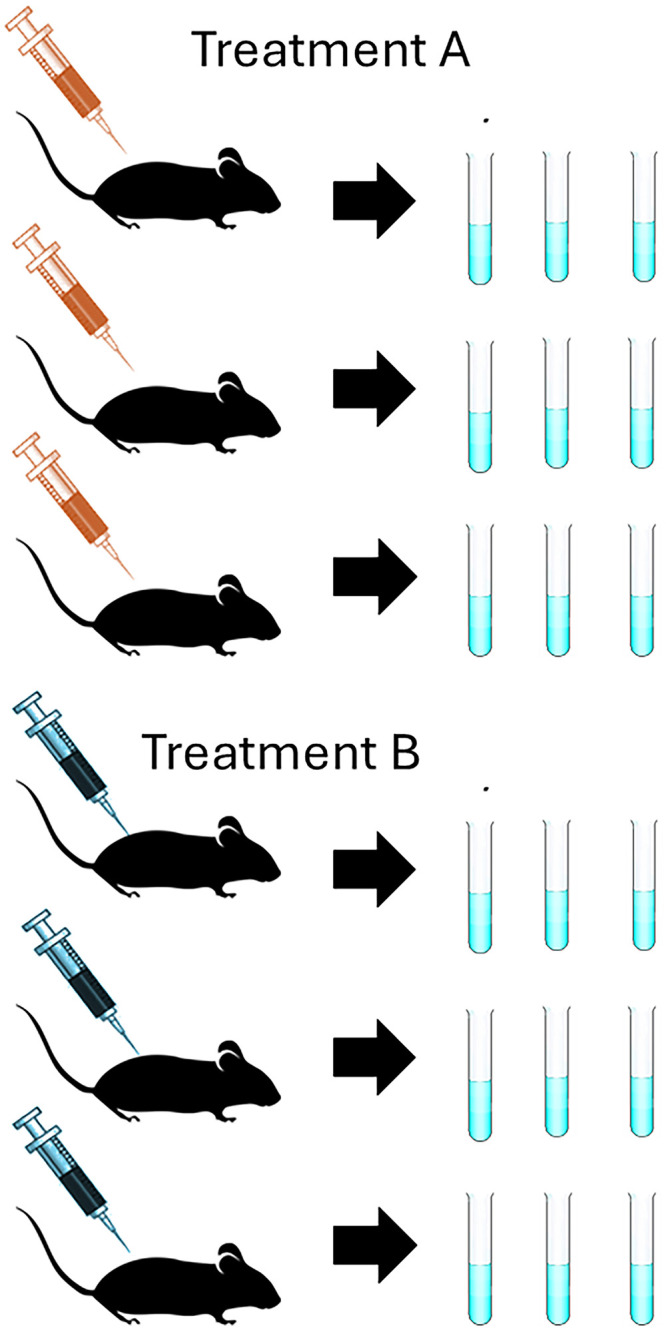
Experimental units, biological units, technical replicates. In this example, six mice are randomized to receive one of two treatments, A or B. The individual mouse is the experimental unit. The total sample size is *N* = 6, with three mice per treatment group. There are *n* = 3 experimental units and *n* = 3 biological units per group. Lysates in three separate aliquots are obtained from each mouse. The technical replicates are the *n* = 3 aliquot subsamples per mouse. Because the mouse is the experimental unit, the total sample size *N* is still 6, not 18. Analyzing the data from the 18 technical replicates as if these are independent experimental units artificially inflates the true sample size (*pseudo-replication*).

Depending on the objectives of the experiment the researcher must also consider *biological* and *technical* replication (Figs. 1, 2; Table 2). Replication of biological units captures biological variability between and within these units. The biological unit BU is not necessarily the same as the experimental unit EU. Depending on how the treatment intervention is applied, the EU can consist of an individual BU, a group of BUs, a sequence of observations on a single BU, or a part of a BU (Blainey et al., 2014; Lazic et al., 2018). Technical replication is essential part of good scientific practice in laboratory science. Technical replicates are used to verify consistency of the measurement process and ensure that experimental signals are not merely artifact. Technical replicates are multiple measurements made on an experimental unit to obtain an estimate of measurement error or the difference between a measured quantity and its true value. Measurement error is an inevitable part of laboratory determinations and results from differences between operators and instruments, instrument drift, subjectivity in determination of measurement landmarks, or faulty calibration. The variance calculated from the multiple measurements estimates the precision, and therefore the repeatability, of the measurement.

**Table 2.** Different varieties of replicate in a hypothetical single-cell gene expression RNA sequencing experiment

	Replicate unit	Replicate type
Animals	Colonies	Biological
	Strains	Biological
	Cohoused groups	Biological
	Sex (M, F)	Biological
	Individuals	Biological
Sample preparation	Organs from animals killed for purpose	Biological
	Methods for dissociating cells from tissue	Technical
	Individual cells	Biological
	Dissociation runs from given tissue sample	Technical
	RNA-seq library construction	Technical
Sequencing	Runs from the library of a given cell	Technical
	Readouts from different transcript molecules	Biological or technical
	Readouts with unique molecular identifier (UMI) from a given transcript molecule	Technical

Designating a given replicate unit as an experimental unit depends on the central hypothesis to be tested and the study design. Adapted from Blainey et al. (2014).

The replication of entire experiments is a separate issue. “Repeating the entire study three times for reproducibility” in most cases is a cultural artifact coming from investigators, not statisticians, and is based on mistaken ideas of statistical methods and terminology (Fitts, 2011). “Reproducibility” cannot be obtained by a low-quality, biased, nonrandomized, and underpowered study, no matter how many times it is repeated. Truly independent replications (different disease models, times, locations, personnel) can assess how well models and constructs will perform over a variety of conditions and provide further evidence of both robustness and translation potential (Karp, 2018). Similar experiments performed by independent laboratories are the gold standard for validation, reproducibility, and generalizability (*external validity*). Unfortunately, several high profile large-scale independent validation studies have been disappointing. In these studies, disparities in results and thus poor external validity are primarily due to poor *internal validity*: omission of randomization and blinding, poor study design, and too-small sample sizes (Errington et al., 2021a,b).

#### Blocking

Variation is a feature, not a bug, of all experiments conducted on biological material. To get the cleanest and least noisy experimental signal possible, this variation must be controlled. Simply increasing the sample size is not a solution. Instead, reducing variation is the key to reducing animal numbers and increasing the reliability of results, which is why Russell and Burch emphasized their importance as central to the 3Rs strategy. The chief statistical method for controlling variation in designed experiments is *blocking*. A blocking factor is a categorical “nuisance” factor identified as a likely source of variation in the response but not of primary interest to the investigator. The experimental units are grouped into homogeneous “blocks” defined by that nuisance factor. Treatments are then randomly allocated to the experimental units within each block. Nonstatistical methods of reducing variation include training personnel up to standard, and standardizing performance, protocol development, and animal care and husbandry practices. Blocked designs and methods of blocking will be described in greater detail in Part 2.

## Summary

Poor study design and incorrect and inappropriate statistical analyses have been estimated to account for over one-half of all reproducibility problems in preclinical research (Freedman et al., 2015) and have been identified as major contributors to well-publicized failures in replicability, reproducibility, and translation (Wold and Tabak, 2021). Best practices for study reliability and reproducibility must be built into the study early, during protocol preparation and planning, and before experiments begin. High quality and humane experimentation requires incorporation of best scientific practices, including both statistical design strategies and nonexperimental features such as experimental logistics and quality control (Smith et al., 2018). Biases resulting from poor study design, lack of randomization and blinding, inappropriate sample sizes, and failure to standardize experimental procedures and processes will distort findings and invalidate conclusions. Researchers have the responsibility of conducting all experiments, and especially those involving animals, to the highest possible standards and ensuring that their data are valid reliable, and scientifically informative. Only then will animal use in research be ethically justifiable.

## Synthesis

Reviewing Editor: Mihaela Iordanova, Concordia University - Loyola Campus Decisions are customarily a result of the Reviewing Editor and the peer reviewers coming together and discussing their recommendations until a consensus is reached. When revisions are invited, a fact-based synthesis statement explaining their decision and outlining what is needed to prepare a revision will be listed below. The following reviewer(s) agreed to reveal their identity: Robert Calin-Jageman.

This manuscript provides a primer on the fundamentals of statistically based design of experiments. This is an important set of topics that can help researchers move beyond the simple two-group design to designs that better control for nuisance variables and which provide higher efficiency in terms of evidence per experimental unit. I feel confident that eNeuro readers will find value in this paper -- they will gain concepts and references that will help them down the path towards better experimental designs and away from some common but troubling issues (e.g. psuedoreplication)

eNeuro is very enthusiastic about this clear, well-organized paper. It gives foundational definitions of the elements of an experimental design and explains how randomization, replication, and blocking can provide causal clarity with maximum efficiency. While every scientists has at least some ideas about experimental design, this clear and concise treatment helps readers understand several common pitfalls (psueoreplication and poor randomization) and helps make clear why graduating from simple two-group designs can help improve clarity and efficiency.

The first draft of this paper was already strong. I provided extensive in-text comments related to style, organization, and clarity. The author has addressed all my comments and concerns. In my judgement, this is ready for publication.
